# Immunogenicity Studies in Carnivores Using a Rabies Virus Construct with a Site-Directed Deletion in the Phosphoprotein

**DOI:** 10.4061/2011/898171

**Published:** 2011-09-21

**Authors:** Ad Vos, Karl-Klaus Conzelmann, Stefan Finke, Thomas Müller, Jens Teifke, Anthony R. Fooks, Andreas Neubert

**Affiliations:** ^1^IDT Biologika GmbH, Am Pharmapark, 06855 Dessau-Rosslau, Germany; ^2^Max-von-Pettenkofer Institut, Ludwig-Maximilians-University, Feodor-Lynen-Straße 25, 81377 Muenchen, Germany; ^3^Institute for Molecular Biology, Friedrich-Loeffler-Institut, Federal Research Institute for Animal Health, Südufer 10, 17493 Greifswald-Insel Riems, Germany; ^4^Institute for Epidemiology, Friedrich-Loeffler-Institut, Federal Research Institute for Animal Health, Seestraße 55, 16868 Wusterhausen, Germany; ^5^Laboratory for Pathology and Bacteriology, Friedrich-Loeffler-Institut, Federal Research Institute for Animal Health, Südufer 10, 17493 Greifswald-Insel Riems, Germany; ^6^Animal Health and Veterinary Laboratories Agency, Wildlife Zoonoses and Vector-Borne Diseases Research Group, Weybridge, New Haw, Addlestone, Surrey KT15 3NB, UK; ^7^The National Centre for Zoonosis Research, University of Liverpool, Leahurst, Chester High Road, Neston CH64 7TE, UK

## Abstract

Different approaches have been applied to develop highly attenuated rabies virus vaccines for oral vaccination of mesocarnivores. One prototype vaccine construct is SAD dIND1, which contains a deletion in the P-gene severely limiting the inhibition of type-1 interferon induction. Immunogenicity studies in foxes and skunks were undertaken to investigate whether this highly attenuated vaccine would be more immunogenic than the parental SAD B19 vaccine strain. In foxes, it was demonstrated that SAD dIND1 protected the animals against a rabies infection after a single oral dose, although virus neutralizing antibody titres were lower than in foxes orally vaccinated with the SAD B19 virus as observed in previous experiments. In contrast, skunks receiving 10^7.5^ FFU SAD dIND1 did not develop virus neutralizing antibodies and were not protected against a subsequent rabies infection.

## 1. Introduction

The European rabies landscape has changed notably in the last 30 years as a result of oral vaccination of foxes (*Vulpes vulpes*) against rabies. Many countries in West and Central Europe eliminated terrestrial wildlife rabies by distributing oral rabies vaccine (ORV) baits. The red fox is no longer the sole target species for those baits; oral rabies vaccination programmes are targeted at many different animal species in many different regions, worldwide. The presently available commercial ORV are all replication-competent viruses and therefore pose an intrinsic safety risk. Vaccine virus-associated rabies cases have been reported in target and nontarget species [1–3]. Also, adverse reactions have been observed in humans after direct exposure to a recombinant ORV [4, 5]. Furthermore, several animal species are difficult to vaccinate against rabies by the oral route using the available products. Hence, an ongoing programme for safer ORV candidates with improved efficacy has been initiated since the first ORVs became commercially available [6]. One group of candidates is based on site-directed mutations of the rabies virus genome. Originally, these efforts focused on the gene encoding the rabies virus glycoprotein. The principal modality of protection against rabies virus is the generation of virus-neutralising antibodies (VNA) against the glycoprotein [6]. However, other facets of innate and adaptive immunity also contribute to virus clearance. Moreover, the role of cell-mediated and humoral immunity against the nucleoprotein, matrix protein, and phosphoprotein in the protection of animals against rabies remains unclear. For these reasons, other rabies virus genes offer possibilities for the development of highly attenuated viruses, for example, the ability of the phosphoprotein to counteract transcriptional activation of interferon (IFN) Type 1 by interfering with the phosphorylation of IRF-3 [7, 8]. A site-directed deletion of a small internal domain (aa 176–186) of the phosphoprotein abolishes the ability to efficiently prevent IRF-3 activation and thereby preventing IFN Type 1 induction [9]. A virus construct, SAD dIND1, was developed comprising the aa 176–181 deletion in the phosphoprotein that had lost most of its inhibitory activity in preventing IRF-3 activation. This construct was shown to be completely avirulent in adult mice after i.c. administration [9], underscoring the type I IFN system as probably the most powerful antiviral response capable of controlling viral infections in the absence of adaptive immunity [10]. A pilot experiment in two wildlife reservoir species was initiated to investigate whether SAD dIND1 is more immunogenic than the parental strain SAD B19. The two wildlife reservoir species assessed included the highly susceptible red fox and the striped skunk (*Mephitis mephitis*); the latter is an animal species notoriously refractory to oral rabies vaccination [11, 12]. Although foxes vaccinated with SAD dIND1 by the oral route survived a subsequent rabies challenge, the construct was not able to protect skunks against a subsequent rabies infection.

## 2. Material and Methods

### 2.1. Virus Construct

The SAD dIND1 construct was developed as previously described [9] using the recombinant rabies virus SAD L16 comprising the consensus sequence of the ORV strain SAD B19. Through site-directed mutagenesis codons specifying the amino acids 176 to 181 of the phosphoprotein were deleted. The virus material used in these studies was propagated using a MOI of 0.1 in BSR T7/5 cells, a BHK-derived cell line stably expressing t7 RNA polymerase [13]. SAD dIND1 is considered a genetically modified organism (GMO) and is classified as a BSL2 pathogen in Germany. The permits for the animal studies with such GMOs have been obtained from the appropriate regulatory authorities (AZ-66230-1501-3, Landesverwaltungsamt Sachsen-Anhalt, Germany). 

### 2.2. Challenge Virus

The challenge virus, CVS/USA/TX Coyote/295/R/061893, was obtained from CDC (USA) and originally isolated from a salivary gland of a coyote (*Canis latrans*). The original challenge virus had been inoculated in a fox and reisolated from the brain after the animal succumbed to rabies. The virus isolate was passaged once in a fox and finally reisolated from the salivary gland. The foxes and skunks were administered 1.0 mL of the challenge virus (10^5.1^ MICLD50) in the M. masseter by the i.m. route.

### 2.3. Animals

The captive bred animals were obtained from different commercial sources; foxes from Fa. Phu Foxpol, Poland, and skunks from AGHIA Birds Company, the Netherlands. The animals were identified by implantation of a microchip (UNO BV, Zevenaar, The Netherlands). Vaccinated foxes were kept individually in cages housed within an isolation unit of the animal house at IDT. Vaccinated skunks were caged in groups within an isolation unit until the time of challenge when each animal was housed individually. The control animals were housed in the outside animal enclosure until administration of the challenge virus when the animals were also transferred to individual cages within an isolation room. Foxes were fed once a day and water was offered ad libitum. Foxes received fresh commercial food for fur animals, (Schirmer & Partner, Doehlen [D]). The skunks were fed commercial dry pet food twice a day (100 g = 25 g cat food (Drei-Mix, Miehlitz KG) + 75 g dog food (Good deal, Voro-Dog Vertrieb, Enger), soaked with water substituted with 5 g vitamin mixture per animal once per day (Multivit, Inropharm GmbH Fürstenzell)). The skunks were also fed fresh fruit daily. The animals were observed at least once a day. After challenge, the animals were observed more frequently, and, on onset of CNS-related clinical signs, the animals were euthanized with T61 intracardial, 0.3 mL per body weight kilogram (Intervet Deutschland GmbH, Unterschleissheim [D]), after sedation with a ketamine 1.0–2.0 mL 10% (WDT eG, Garbsen [D])-xylazine [Xylariem, Riemser Arzneimittel, Riems [D]) mixture. 

The housing conditions of the animals met the conditions as stated in the German animal welfare act §2&2a and the recommendations of the GV-SOLAS (Society for laboratory animal science). All experiments were undertaken in accordance with the German animal welfare act §8a, Abs. 1 and 2.f.

### 2.4. Vaccination

Eight adult foxes were vaccinated with 1.5 mL SAD dIND1 using different doses and routes of administration (Table 1). The animals were challenged together with 2 control animals 62 days after administration of SAD dIND1. Fifty days after challenge, all surviving animals were euthanized. Blood samples were taken on day 0 (prior to vaccination), 28, 58, and 112 after vaccination. During a dose-dependent study with different vaccine candidates, 3 skunks received 10^7.5^ FFU SAD dIND1 by direct oral instillation and were challenged 45 days later together with 2 control animals. Blood samples of the skunks were collected—5, 28, 42, and 57 days after vaccination. All animals were sedated during administration of SAD dIND1 and blood sampling.

### 2.5. Assays

#### 2.5.1. FAT/IHC

The presence of rabies virus antigen in fox brain samples was examined using the Fluorescent Antibody Test (FAT) as described by Dean et al. [14]. Samples of hippocampus from skunks were fixed in 4% phosphate-buffered formaldehyde and processed for paraffin-wax embedding and immunohistochemistry (IHC), slightly modifying a method described previously [15]. The hippocampus of skunks has been shown to be highly suitable for these purposes [16].

#### 2.5.2. RFFIT

Blood samples collected were examined for the presence of rabies VNA using the rapid fluorescent focus inhibition test (RFFIT) [17], with the modifications of that method as described by Cox and Schneider [18]. Prior to testing, serum samples were heat-inactivated for 30 minutes at 56°C. To calculate the titer, a 50% reduction in concentration of rabies virus in vitro was calculated by use of inverse interpolation (SAS software 9.2). The rabies VNA titers were converted to international units (IU) by comparison with international standard immunoglobulin adjusted to 0.5 U/mL, which served as a positive control.

## 3. Results

All foxes vaccinated with SAD dIND1 survived the lethal challenge irrespective of dose and route of administration and were euthanized 50 days after challenge. Meanwhile, both control animals were euthanized upon showing clinical signs to rabies infection 10 and 11 days after challenge. Rabies antigen was detected in brain material of both animals. All foxes receiving SAD dIND1 seroconverted with VNA titres greater than the arbitrary threshold of 0.5 IU/mL (Table 2). The level of rabies VNA antibodies indicated a dose-and route-dependent response. In contrast to the foxes, SAD dIND1 did not confer sufficient immunity in skunks to prevent infection after challenge exposure. Unfortunately, the small sample size of skunks was even further reduced. One skunk receiving SAD dIND1 was excluded from challenge because it developed a severe dermatitis during the observation period and was located to another isolation unit. All 4 remaining animals succumbed to rabies as confirmed by IHC. The two control animals and the two vaccinated animals were euthanized upon showing clinical signs of CNS disorders on day 13 after challenge. None of the skunks that received SAD dIND1 developed detectable levels of rabies VNA during the observation period (Table 3). After challenge, both control animals had detectable levels of VNA, meanwhile both animals that received SAD dIND1 remained seronegative.

## 4. Discussion

One of the major concerns with the distribution of ORVs for wildlife in the environment is the risk that nontarget species, including humans, will contact these vaccine viruses. The safety concerns associated with the first generation attenuated ORV led to the development of different recombinant vaccine vectors expressing the rabies virus glycoprotein; for example, vaccinia virus, human adenovirus type 5, pseudorabies virus, canine herpesvirus, and canine adenovirus type 2 constructs [19–24]. Some of these replication competent vectors are based on human pathogens and are therefore also not without risks, especially in view of immunocompromised persons. Unfortunately, replication-deficient constructs like an E1-deleted human adenovirus type-5 expressing the rabies virus glycoprotein did not induce detectable rabies VNA after oral administration [25]. The balance that must be attained is constructing a viral delivery system that is fully attenuated to render it safe and prevent replication and yet have sufficient viral characteristics that allow uptake into permissive cells and protein production to induce an immune response. A further important limitation of some of these recombinant constructs is the interference of preexisting immunity to the vector virus, severely compromising its efficacy [26–28]. Preexisting immunity to the vector virus would not be important for highly attenuated rabies virus constructs. Therefore, several rabies virus constructs have been selected or developed and tested for their potential use as a rabies vaccine, including constructs with site-directed deletions in the phosphoprotein or its complete deletion [29–31]. For example, removal of Dynein Light Chain 8 binding site motif substantially reduced viral transcription and replication in the central nervous system [32]. Another strategy was to make the expression of the essential phosphoprotein dependent on translation and not transcription [33]. The SAD dIND1 construct uses a different approach which is aimed at inducing an improved innate immune response in vaccinated animals. The deletion introduced by site-directed mutagenesis interferes with the virus countermeasures to inhibit induction of IFN [34]. The enhanced antiviral host response was demonstrated in mice that were inoculated i.c. with SAD dIND1 or with the parental strain SAD L16. While all mice inoculated with SAD L16 succumbed to rabies, all SAD dIND1 inoculated animals survived [9]. An oral vaccine candidate must be safe and efficacious, preferably conferring life-long immunity, after the consumption of a single bait [35]. Although all foxes vaccinated with SAD dIND1 were fully protected against the severe rabies virus challenge, the VNA was lower than observed in foxes offered a bait containing 10^6.3^ FFU of the vaccine strain SAD B19 [36]. The geometric mean titre of 27 foxes was 43.5 and 33.9 IU/mL 60 and 110 days after the animals had consumed a SAD B19 vaccine bait, respectively. The difference in VNA titre is more notable considering that the administration of the SAD B19 vaccine was by bait consumption instead of by direct oral gavage for SAD dIND1. The lower VNA titres observed in the SAD dIND1 vaccinated animals could have been a result of the IFN Type 1 induced shift towards a Th1 immune response [7]. However, it is more probably the reduced viral growth and antigenic presentation of the rabies virus glycoprotein to antigen presenting cells. An increased induction of IFN by SAD dIND-infected cells would result in limited viral spread because noninfected neighbouring cells have been placed into an “antiviral” state by expression of antiviral IFN stimulated genes through the IFN-signalling pathway [10]. Rabies virus released from primary infected cells replicate inefficiently in such cells. Unfortunately, SAD dIND1 failed to elicit detectable levels of VNA in skunks, and consequently none of the animals induced a protective immune response against the challenge. The reduced ability of SAD dIND1 to induce rabies VNA compared to the SAD B19 vaccine strain was also shown in skunks. During a previous safety study with SAD B19, 3 of seven skunks receiving10^7.9^ FFU by direct oral gavage seroconverted, and all three animals had measurable levels of rabies virus VNA (>5.0 IU/mL) 296 days after vaccination [37]. From this study, it can be concluded that the enhanced IFN production in response to SAD dIND1 results in a strong antiviral effect that outperforms the acknowledged immune-stimulatory effect of type I IFN. In order to make the highly attenuated replication competent virus, SAD dIND, an effective rabies vaccine candidate for oral vaccination of species including the striped skunk its immunogenicity must be improved. This could be achieved by altering the deleted domain, whereby the inhibitory effect on IRF-3 activation can be adjusted. For example, another construct (SAD dIND2) with a phosphoprotein lacking amino acids 182–186 instead of 176–181 (SAD dIND1) inhibits IRF-3 activation less efficiently [9]. Therefore, it can be assumed that, in SAD dIND2 infected hosts, there is less IFN Type 1 induction subsequently leading to more pronounced viral spread and antigenic presentation. However, SAD dIND2 was in contrast to SAD dIND1 not completely avirulent in mice [9], once more underscoring the difficulties in determining the delicate balance between safety and efficacy.

## Figures and Tables

**Table 1:** Summary of the experimental design with SAD dIND1 in foxes and skunks (o.g.: oral gavage; i.m: intramuscularly).

Animal	Number	Dose FFU/mL	Route	Observation period (days)
Fox	3	10^6.3^	o.g.	62
Fox	3	10^7.3^	o.g.	62
Fox	2	10^6.3^	i.m.	62
Skunk	3	10^7.5^	o.g.	45

**Table 2:** The titre of virus neutralizing antibodies measured in the fox blood samples by RFFIT are expressed in IU/mL. The final blood sample (B3) was taken 50 days after challenge (GMT; geometric mean titre; o.g: oral gavage; i.m: intramuscularly).

Animal	Route	Dose FFU/mL (10 log)	B1 (day 28)	B2 (day 58)	B3 (day 112)
7879	o.g.	7.3	47.5	23.8	80.0
0200	o.g.	7.3	11.9	47.5	80.0
9787	o.g.	7.3	56.6	11.9	47.5

GMT			31.7	23.8	67.2

6170	o.g.	6.3	14.2	14.2	40.0
7287	o.g.	6.3	11.9	11.9	80.0
9788	o.g.	6.3	14.2	10.0	20.0

GMT			13.4	11.9	40.0

3568	i.m.	6.3	23.8	23.8	56.6
6061	i.m.	6.3	28.3	23.8	91.9

GMT			26.0	23.8	72.1

**Table 3:** The results of the blood samples from the skunks, including the control animals; the titre of the virus neutralizing antibodies are expressed in IU/mL and determined by RFFIT (n.d: not determined).

Animal	Construct	B0 (day 5)	B1 (day 28)	B2 (day 42)	B3 (day 57)
9892	SAD dIND1	0.04	0.03	0.03	0.15
9893*	SAD dIND1	0.09	0.06	0.09	n.d.
9895	SAD dIND1	0.11	0.06	0.06	0.11
9899	control	0.06	n.d	n.d	3.44
9897	control	0.05	n.d	n.d	4.31

*Animal developed severe dermatitis and was excluded for challenge.
